# Genetic mechanisms for estuarine carbohydrate degradation and linked transcriptional activity

**DOI:** 10.1128/aem.01852-25

**Published:** 2026-01-13

**Authors:** Jian Sheng Boey, Hwee Sze Tee, David W. Waite, Kim M. Handley

**Affiliations:** 1 School of Biological Sciences, University of Auckland 1415, Auckland, New Zealand; Royal Botanic Gardens Kew, Surrey, United Kingdom

**Keywords:** carbohydrates, CAZymes, estuary

## Abstract

The current understanding of carbohydrate substrate degradation is largely derived from incubation experiments involving specific substrates. In estuaries, carbohydrates are often grouped together with other sources of carbon, for analytical purposes, and measured as total and fractional organic matter. Here, we describe putative carbohydrate degradation at the polysaccharide level by the prokaryotic community in an estuary. Samples were collected along a freshwater-to-marine salinity gradient from both the water column and underlying benthic sediments. Metagenomic and metatranscriptomic data were used to determine carbohydrate-active enzyme (CAZyme)-encoding metagenome-assembled genomes and associated transcriptional activity across the gradient. Previous work demonstrated assimilation of xylan (a component of hemicellulose) in estuaries. We show the genetic mechanisms associated with the degradation of xylan, as well as arabinogalactan (also from hemicellulose), and various other glycans were widespread among estuarine taxa and actively expressed. In addition, results show different carbohydrate degradation strategies between planktonic and benthic organisms. For example, results indicate that sediment communities harbored a greater variety and density of CAZyme-encoding genes and capacity to degrade complex plant biomass (cellulose and hemicellulose) and dedicated more gene transcription overall to CAZymes than planktonic communities. In contrast, planktonic prokaryotes tended to express a greater fraction of their CAZyme-encoding gene repertoires. The transcription of gene clusters associated with degrading beta-1,3-glucans such as laminarin was prevalent in the water column. Microbial activity to degrade alpha-glucans such as glycogen was predicted to be ubiquitous but was greatest in planktonic communities. Taken together, results highlight differences in the capacity of planktonic and benthic communities to degrade carbohydrates, which reflect differences in substrate availability and complexity.

**IMPORTANCE:**

Estuaries are productive ecosystems that combine various forms of organic carbon from autochthonous (e.g., algal primary producers and mangroves) and allochthonous (e.g., terrestrial plant) sources. The degradation and recycling of this organic carbon is driven by heterotrophic bacteria that are expected to harbor diverse genetic mechanisms for carbohydrate degradation to match the diversity and complexity of organic carbon encountered in the environment. Results here illustrate the diversity of carbohydrate-active enzymes (notably glycosyl hydrolases) encoded by estuarine communities and the different substrate prioritizations of planktonic and benthic communities.

## INTRODUCTION

Estuaries cover just 0.2%–0.3% of the Earth’s oceanic area, yet their carbon cycling capacity outperforms that of many other environments (1, 2). As intermediaries between riverine and marine habitats, estuaries receive nutrients that are typically limited in either of these other adjacent habitats (e.g., phosphates in freshwater; nitrates in seawater) (3). The mixing of these nutrients in estuaries can lead to high levels of microbial primary productivity (3). A major product of primary production is the carbohydrates that form cell walls or store energy, which account for 18%–37% of dissolved organic carbon in the system (4, 5). Much of this organic carbon, along with allochthonous carbon, fuels heterotrophic respiration, resulting in high productivity (6, 7).

Most autochthonous organic carbon within estuaries is typically derived from photosynthetic microeukaryotes (8, 9) and, where present, mangroves (8). Both phytoplankton and microphytobenthos can contribute to estuarine primary production *in situ* (7, 10), although phytoplankton can be referred to as allochthonous due to tidal transport (9). Carbohydrates comprising this microbial fraction are dominated by simple sugars, starches, and exopolymers, along with other substrates, for example, laminarin produced by microalgae (11, 12). Additional organic carbon may be derived from macroalgal biomass (13), with substrates including ulvan from green algae (14, 15) and fucoidan, laminarin, and alginate from brown algae (15, 16). Allochthonous carbohydrates in estuaries are primarily derived from inputs of vascular plant biomass (e.g., lignocellulose and hemicellulose such as xylan) (17). This plant biomass is typically more recalcitrant to degradation compared to autochthonous carbohydrates due to factors including structural complexity, cellulose crystallinity, hemicellulose composition, and lignin content. The distribution and composition of both autochthonous and allochthonous carbohydrates co-vary with the geography, which coincides with salinity (4, 5) and proximity to plants such as mangroves (8), and with temporal changes such as phytoplankton blooms (18).

With the exception of some recalcitrant carbohydrates, other polysaccharides and labile carbohydrate monomers can be rapidly degraded by microbial communities in freshwater (19) and marine (20, 21) environments, and their degradation products fuel estuarine biogeochemical cycles (22). The biosynthesis, degradation, and transformation of carbohydrate substrates are catalyzed by carbohydrate-active enzymes (CAZymes) (15). CAZymes encoded by prokaryotes for polysaccharide degradation are diverse (23, 24) and phylogenetically widespread (24). Over 80% of prokaryotes with sequenced genomes are predicted to degrade the storage polysaccharides starch and glycogen, and over half are predicted to degrade structural polysaccharides (24). CAZymes are organized into six superfamilies, namely, glycosyl hydrolases (GH), glycosyltransferases (GT), polysaccharide lyases (PL), carbohydrate esterases (CE), carbohydrate-binding modules (CBM), and most recently, auxiliary activities (AA) (25). All CAZyme superfamilies, except GT, are involved in substrate degradation. GH and PL (the primary superfamilies of interest for this study) cleave glycosidic bonds aided by CE for removing alkyl groups and CBM for substrate recognition and binding. The degradation of structurally complex polysaccharides requires a suite of CAZymes that operate synergistically. For instance, members of Bacteroidota and Verrucomicrobiota transcribe multiple CAZymes to degrade algal fucoidan and ulvan (26, 27) and pectic rhamnogalacturonan II (28). Recalcitrant substrates such as crystalline cellulose require CAZymes (GH, PL, and CE) and other structural motifs (cohesin and dockerin) organized as a cellulosome for efficient degradation (29, 30).

Carbohydrate degradation is an area of active and intense research, but much of this has concentrated on fungi and host-associated prokaryotes. Environments such as the guts of humans (31, 32), other mammals (33, 34), fish (35), and insects (36, 37) are well suited for this area of research given their constrained substrate pools and microbiomes. Fundamental research and discoveries made in this area have contributed to advances in biotechnology (38, 39) and medical sciences (40). In open environments, carbohydrates—and their degradation—are often bundled into measurements of dissolved and particulate organic carbon for analytical convenience (15), although not always (41). These measurements are used as parameters of ecosystem-wide functions such as respiration (42) and primary production (7, 43). When carbohydrates are explicitly measured, they are often reported based on their extraction method (hot water versus acid) (44) or size/length (monosaccharide versus polysaccharide) (19, 45). This simplification is necessary given the structural complexity and variety of oligo- and polysaccharides. Studies now also leverage multi-omic techniques to characterize the metabolic capacity and potential for carbohydrate degradation in the environment. Indeed, valuable insights on microbial lifestyle strategies (46, 47), host adaptations (48), ecological niches (49), and metabolic transformations (50) can be obtained by proxy from metagenome-assembled genomes (MAGs) and associated CAZyme-encoding genes.

In this study, we characterized the spatial and taxonomic distribution of CAZyme-encoding genes and transcriptional activity across the freshwater-brackish-marine salinity gradient of a tidal lagoon estuary. Water and sediment samples were collected from the subtidal zone and were used to generate metagenomes and metatranscriptomes. CAZymes encoded by estuarine taxa were determined across all six classes (i.e., AA, CBM, CE, GH, GT, and PL), and the suite of carbohydrate substrates that could be degraded by these CAZymes were predicted. Results indicate diverse and synergistic mechanisms are required for the utilization of carbohydrate substrates, of varying complexity, that are predicted to change substantially in composition across the estuary and between the water column and benthic habitats.

## MATERIALS AND METHODS

### Sample collection, site description, and data generation

Sample collection, geochemistry, and sequence data generation and assembly are described in detail by Tee et al. (10) and summarized here. Sediment and water samples were collected from nine subtidal sites along the salinity gradient of the Waiwera river and estuary (Auckland, New Zealand) (Fig. S1). The estuary is a shallow tidal lagoon estuary that is permanently open, with mangroves (*Avicennia marina*) intermittently present along the brackish intertidal zone. Sampling site salinities ranged from fresh (sites 1 and 2, average salinity of 0.3 ppt) to brackish (sites 3 to 7, average salinity of 19.8 ppt) to marine salinities (sites 8 and 9, average salinity of 30.2 ppt). Sediments were collected in triplicate (approximately 5 m apart, top 2 cm). Individual water samples (of ≥ 10 L) were collected per site at a depth of approximately 0.2 to 1.0 m, with biomass collected on 0.22 μm pore size filters. The river upstream of sampling sites passed through rural land, and vegetation on either side of the river at sampling sites comprised a diverse mix of plants, including trees and grass (see Tee et al. [10] for site photos), and as noted above mangroves were present along the brackish estuarine transect. The two marine sites were at the estuary mouth with sandy beach either side. No subtidal or intertidal macroalgal growth was visible anywhere along the estuarine transect. However, based on our previous genomic and transcriptomic analyses, both phytoplankton and microphytobenthos were present and metabolically active at saline sites (10).

DNA and RNA were co-extracted from each sample for metagenomics and metatranscriptomics. RNA from sediment at sites 8 and 9 was excluded from further analysis due to low quality (i.e., the lack of clear 23S and 16S rRNA gene peaks in bioanalyzer electropherograms, as reported previously [10]). RNA integrity numbers (RIN) for the remaining samples were on average 6.9 ± 0.4 SD and ranged from 6.0 to 7.8 (except for the site 3 water sample for which no RIN was obtained, but clear rRNA gene peaks were observed). DNA and ribosomal RNA-depleted RNA were sequenced using the Illumina HiSeq platform. For metatranscriptomic data, residual rRNA reads were removed using SortMeRNA v2.1 (51). Paired reads were adapter- and quality-trimmed (minimum Phred score = 30), retaining RNA reads 60–125 bp and DNA reads 80–250 bp.

### Generation of MAGs

Trimmed DNA reads were assembled using metaSPAdes (52), as described by Tee et al. (10), with individual assemblies undertaken of water samples from each site (*n* = 9 assemblies) and co-assemblies undertaken of triplicate sediment samples per site (*n* = 9 co-assemblies). MAGs were generated as described by West et al. (53), and as outlined here. Contigs (> 2 kb) from each assembly were binned using CONCOCT v0.4.1 (54), MaxBin v2.2.4 (55), and MetaBAT v2.12.1 (56), with coverage information determined by mapping sample reads to their corresponding assemblies using BBMap v37.93 with default settings (57). The best MAGs across binning tools per assembly were selected using DAS_Tool v1.1.1 (58), resulting in a set of 1,084 MAGs. MAGs were quality-checked using CheckM v1.2.1 (59), and their taxonomy was predicted using GTDB-Tk v2.4 (60). Genomes with ≥ 50% completeness and ≤ 5% contamination (after accounting for strain heterogeneity) were retained for downstream analyses (*n* = 702).

### Identification of CAZyme-encoding genes and associated enzymatic function

To obtain a set of putative CAZyme-encoding genes, predicted protein sequences were determined by Prodigal v2.6.3 (61). These were searched against HMM profiles in dbCAN HMMdb v13 (62) using hmmsearch from HMMER v3.3.2 (63), with the -z flag set to the HMM profile database size. Domain hits were then parsed using a modified hmmscan-parser.sh script to obtain non-overlapping domain matches and then filtered to retain matches with E-values < 1 × 10^−18^ and domain coverage ≥ 35%. Genes with valid hits after filtering were then assigned CAZy family/families based on the matching profiles (note: more than one CAZy family can be assigned to a gene).

Enzyme commission (EC) numbers are essential for performing fine-grained functional analysis and substrate prediction (see the next sub-section) based on putative CAZyme-encoding genes. EC assignments were obtained by matching annotations from several database searches. First, additional searches were made against the dbCAN-sub database (using hmmsearch) and CAZyDB database (using DIAMOND protein searches) available in dbCAN3 (62). Both dbCAN-sub and some sequences in CAZyDB contain EC numbers for characterized CAZymes. Domain matches from dbCAN-sub were parsed and filtered according to the same criteria as for dbCAN HMMdb v13, whereas DIAMOND matches against CAZyDB were filtered to retain hits with E-value < 1 × 10^−102^ (62), percent ID ≥ 35%, and query coverage ≥ 70% (64). Then, EC numbers were assigned by considering these, and Kyoto Encyclopedia of Genes and Genomes (KEGG) annotations described in the section above, and applying the following rules in order.

- For CAZyme-encoding genes with dbCAN-sub hits, EC numbers were taken from the dbCAN-sub sub-family metadata if available.
- For CAZyme-encoding genes that did not meet criteria 1, where KO annotations were assigned by DRAM with EC numbers in their metadata, these EC numbers were checked against those associated with the gene’s CAZy family annotation. EC numbers were assigned if both annotations agreed.
- If CAZyme-encoding genes did not satisfy criteria 1 and 2, EC numbers were assigned if hits to CAZyDB contained EC numbers and were the same CAZy family.
- For remaining CAZyme-encoding genes, where KEGG annotations (assigned by BLASTp) had EC numbers in their metadata, these EC numbers were checked against those associated with the gene’s CAZy family annotation. EC numbers were assigned if both annotations agreed.

CAZyme-encoding genes that did not satisfy any of the above criteria were not assigned EC numbers. Our method of assigning EC numbers to CAZyme-encoding genes included databases outside of dbCAN and CAZy, thus allowing for interrogation of function for genes that may share distant homology but are yet to be included in the CAZyDB and dbCAN databases.

CAZyme-encoding genes with assigned EC numbers were used for substrate prediction. EC numbers and CAZy families were matched against substrates curated by the dbCAN3 team with further manual curation to ensure EC numbers assigned based on KEGG orthologies were also included in substrate prediction.

### Identification of CAZyme-gene clusters

CAZymes often function synergistically with each other and with transcription factors, signal transduction proteins, and transporters. These genes are often arranged in an operon-like structure called a CAZyme-gene cluster (CGC; also called a polysaccharide utilization loci, PUL, in Bacteroidota). Here, we define CGCs as contiguous genomic regions that consist of at least one CAZyme-encoding gene and at least one other signature gene (i.e., transcription factor, signal transduction protein, and/or transporter) distanced at most two genes apart. Transcription factors and signal transduction proteins were identified by searching protein sequences against HMM profile databases in dbCAN3, whereas transporters were identified by searching protein sequences against the Transporter Classification Database (TCDB; using DIAMOND) and domain-based characterization of families in TCDB (tcDoms; using HMMER) (65).

Putative substrates for CGCs were predicted using methods (B) and (C) described previously (62). Briefly, method (B) involved a DIAMOND search of protein sequences in CGCs against the PUL database. Query-subject pairs with E-values lower than 1 × 10^−102^ were retained. CGC-PUL pairs were ranked based on summed query-subject bitscores, where the best-matched PUL was the one with the highest summed bitscore that also contained matches to at least one CAZyme and one other signature gene in the subject PUL. Method (C) used gene-wise substrate predictions (described above), where the substrate was chosen based on majority voting of putative substrates assigned to each gene.

### MAG environmental distributions and transcription

To obtain a set of unique MAGs for genome coverage and transcription estimates, we dereplicated MAGs across assemblies using dRep v3.4.2 (66) at 98% average nucleotide identity. Per-sample genome coverage/transcription was determined by mapping metagenomic and metatranscriptomic reads against the resulting 372 unique and quality-filtered MAGs using Bowtie2 v2.5.4 (67) with –sensitive mode. Contig coverage depth and breadth were enumerated using the jgi_summarize_bam_contig_depths script in MetaBAT2 v2.15 (56) and CoverM (68), respectively. A population was considered present in a sample if reads covered ≥ 10% of its MAG. Transcript counts were obtained using the featureCounts function of Subread v2.0.7 (69) with the –countReadPairs flag. Where there were fewer than five mapped read-pairs per gene, these were excluded prior to normalization of transcript count data to transcripts per million (TPM) (70).

### Data analysis

Graphical and statistical analyses were performed in R v. 4.5.1 (71). Heatmaps and other plots (violin, bar, scatter, volcano, and gene maps) were generated using the ggplot2 v3.5.2 package (72). Ordinations with/without Procrustes analysis were based on Bray Curtis dissimilarities and were generated using the vegan v. 2.7-1 package (73). Inverse Simpson’s indices were calculated using the diversity function in vegan. Differential gene expression analysis used three packages for comparison: edgeR v. 4.6.3 (74), DESeq2 v. 1.48.1 (75), and limma v. 3.64.3 (76). Wilcoxon tests were conducted using the R wilcox.test function. Spearman’s correlations were determined using the R cor and cor.test functions. Maps of sampling locations were generated as follows. For the country map, the map_data function in ggplot2 was used with the "world" map data set and subsetted by latitude and longitude coordinates. For the estuary map, an outline was drawn based on an initial map that was generated using the leaflet v. 2.2.3 package (77) with the "Esri.WorldTopoMap" map data set and with the study sampling coordinates supplied (see Tee et al. [10] for all coordinates).

## RESULTS AND DISCUSSION

### CAZyme genes in sediment prokaryotic populations are highly diverse and actively transcribed

Across the estuarine freshwater-to-marine salinity transect (two non-saline, five brackish, and two marine sites), we obtained 702 MAGs, from the original 1,084, that met our quality criteria. These 702 MAGs were recovered from both the benthic sediment (*n* = 373) and overlying water column (*n* = 329) and from 21 phyla (Table S1). CAZyme-encoding genes (CAZyme genes) were identified in all 702 MAGs. From these MAGs, we detected 40,067 putative CAZyme-encoding genes assigned to 310 CAZyme families across all six CAZyme classes (i.e., AA; CBM; CE; GH; GT; and PL). Carbohydrate degradation is mainly catalyzed by CAZymes in the GH and PL classes (23, 78). Accordingly, we recovered a diverse range of degradative CAZyme genes, where 44% of CAZyme genes were assigned to 139 GH and 32 PL families, which constitutes about 70% of all known GH and PL families. Our curation inferred putative functions via EC numbers for 17,368 CAZyme genes, of which 12,642 could be matched to at least one substrate across 45 substrate categories (Table S2). These genes putatively target β-glucans (e.g., cellulose derived from plants and laminarin from algae), α-glucans (e.g., starch from plants, glycogen from animals, and dextran from bacteria), pectic substances (e.g., poly- and rhamnogalacturonans from plants), hemicellulose (e.g., xylan, xyloglucan, β-mannan, and arabinan), algal glycans (e.g., ulvan, alginate, and agarose), and fungal and animal glycans (e.g., N-glycans, α-mannans, and sialic acids) (15, 79, 80).

**Fig 1:**
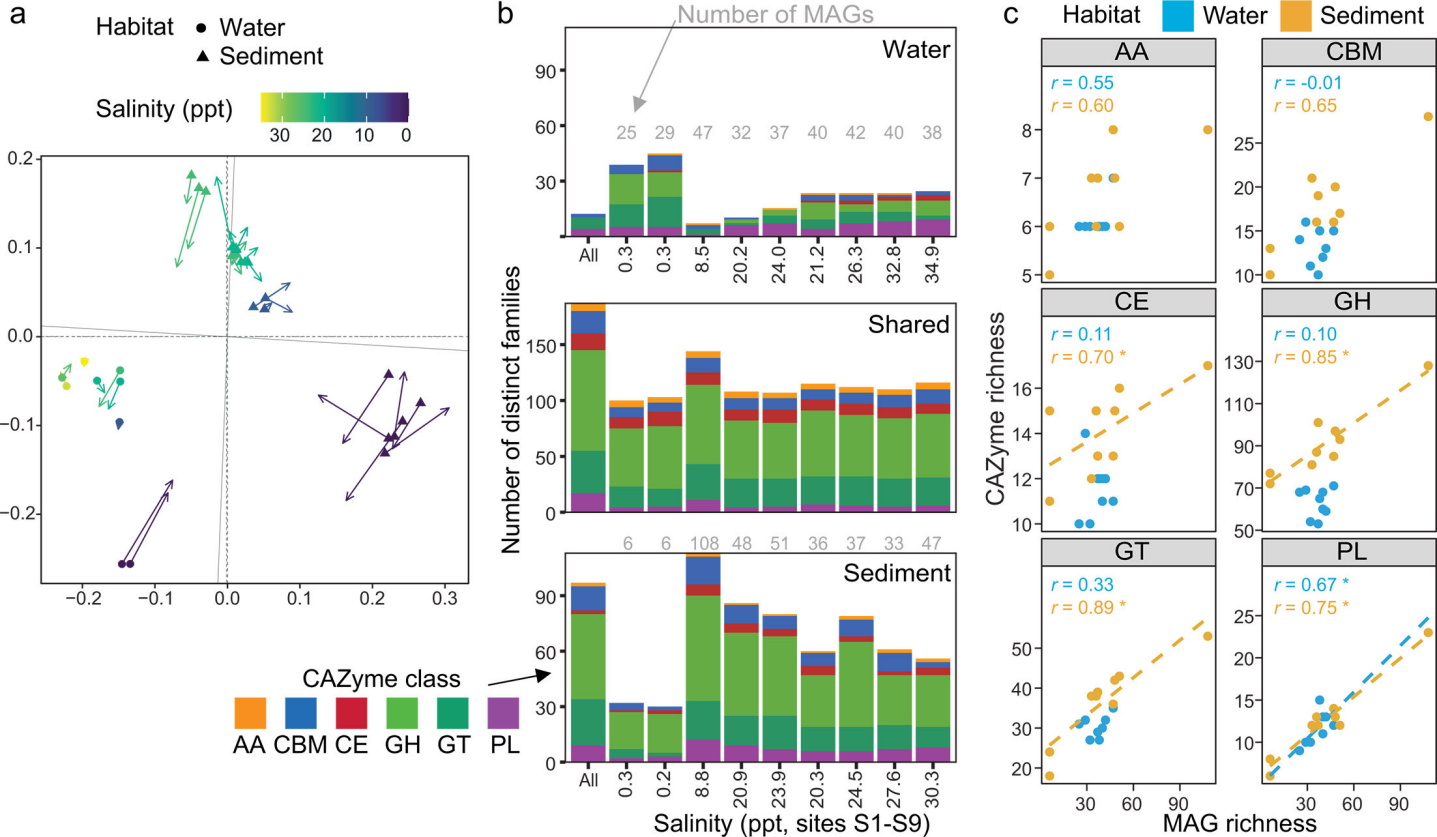
CAZyme composition of estuarine water and sediment. (**a**) Non-metric multidimensional scaling ordination of CAZyme gene Bray-Curtis dissimilarities with procrustean projection of a transcript-based ordination. The ordination for transcripts is projected onto the ordination space of genes. Solid symbols (circles and triangles) represent samples based on CAZyme gene data in the ordination, and arrows (lines and arrowheads) indicate the relative position of the same sample based on CAZyme gene transcripts. No transcriptomes were obtained from marine sediment samples. (**b**) CAZyme richness (i.e., number of distinct families) unique to water or sediment or shared across habitats. Numbers on top of the bars indicate MAG richness (i.e., the number of MAGs recovered from the sample that had CAZyme-encoding genes). Samples along the horizontal axis are ordered from the first non-saline site 1 to the most saline site 9 from left to right. (**c**) CAZyme gene richness against MAG richness by habitat and CAZyme class. Numbers in blue and yellow indicate Spearman correlation coefficients for water and sediment, respectively, and asterisks next to numbers indicate significant correlations (*P*-value < 0.05). Where correlations are significant, linear models are also shown by dashed lines.

CAZyme gene and transcript compositions were strongly delineated by habitat (water versus sediment) and salinity (non-saline versus saline) (Fig. 1a), consistent with differences in the overall community composition within the estuary (10). While the majority of the estuarine CAZyme gene repertoire was found in MAGs recovered from both sediment and water column communities, the sediment community was typically more CAZyme gene (and family)-rich compared to the water column community and despite broadly similar numbers of MAGs across most sites (Fig. 1b). We identified, on average, 18.6 CAZyme genes per MAG in water versus 30.5 in sediment, indicating a higher density of CAZyme encoding in most sediment communities. Non-saline samples represented an exception where comparable CAZyme richness was observed between sediment and water-column communities. This can be attributed to the much smaller number of MAGs recovered from non-saline sediments, relative to saline sediments (and non-saline water), rather than a lower density of CAZyme genes. Accordingly, we found that CAZyme gene richness was positively correlated with MAG richness for four out of five CAZyme classes in sediment, but just one class (PL) in water where there was comparatively little variation in MAG richness (Fig. 1c). Taken together, while taxa and functional richness can be tightly linked (81), the relationship between these only partly explains the carbohydrate degradation capacity identified in this study.

Glycoside hydrolases showed the greatest distinction between habitat types and accounted for most of the differences in CAZyme richness (number of distinct CAZy families harbored by MAGs) between sediment and water communities (Fig. 1c). Despite the general trend for increased CAZyme class richness with MAG richness, GH genes were richer in sediment communities even when comparing water and sediment samples with similar MAG richness, indicating, as for CAZyme genes overall, that GH enrichment in sediments was somewhat independent of MAG or taxa richness. CAZymes from the GH class are central to carbohydrate degradation in any habitat, where the breakdown of carbohydrate substrates must involve members of this CAZyme class at some point along the degradative metabolic pathway (78, 82). To date, GH also constitute the largest CAZyme class (i.e., have the greatest number of families) with the most diverse enzymatic activity. The elevated GH richness observed here suggests that sediment communities are (i) more able to degrade diverse substrates that may not be degradable by water column communities (i.e., greater niche partitioning capacity, which is also indicated by greater numbers of distinct GH family genes in sediment communities, Fig. 1b), and/or (ii) are more able to compete for substrates due to redundancy in GH-encoding genes.

**Fig 2:**
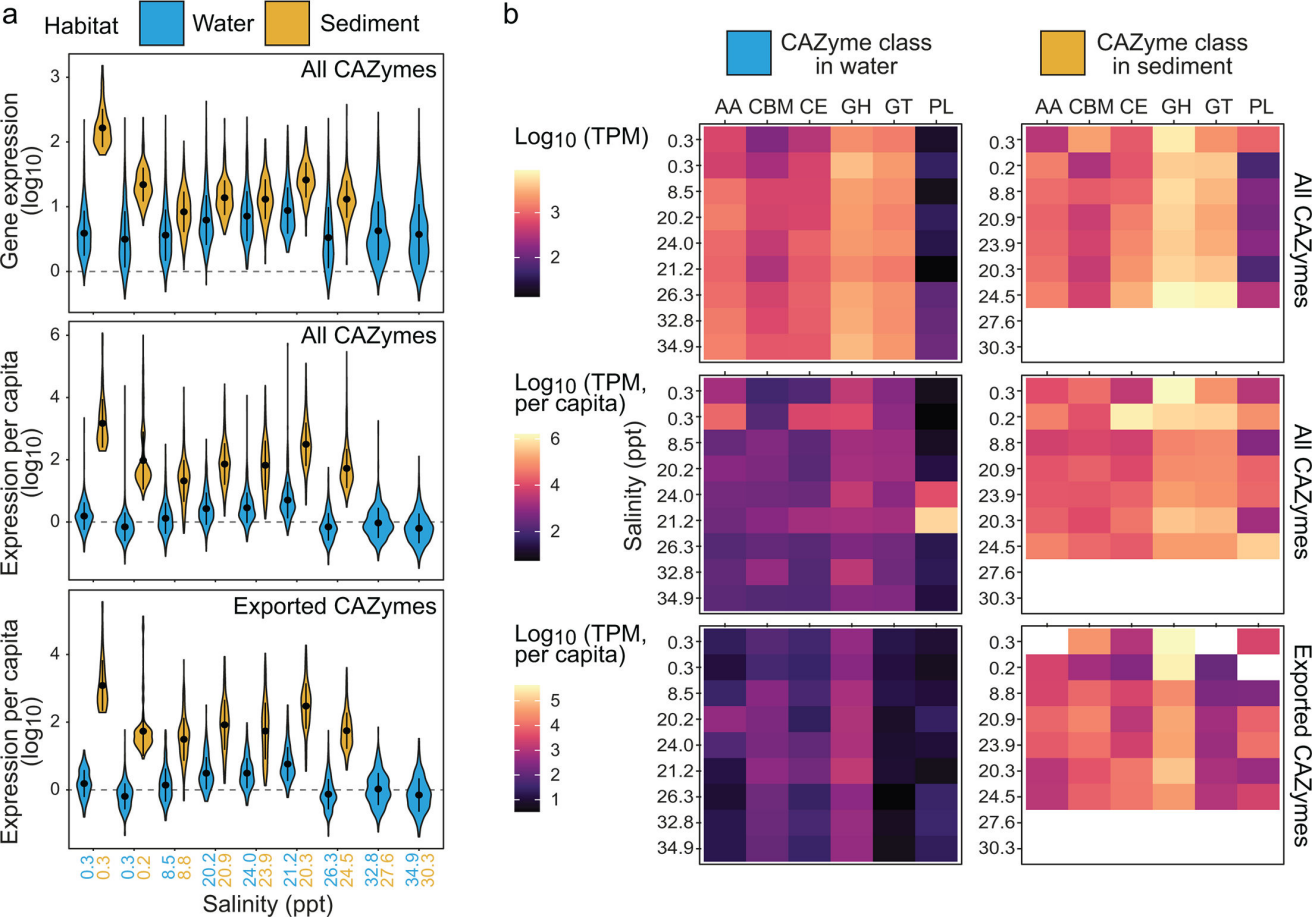
Expression of CAZyme genes across the estuary. (**a**) Distribution of summed gene expression (transcripts per million, TPM) in the estuary overall (top) and after normalizing to genome coverage (expression per capita) for all genes (middle) and the genes of exported CAZymes (bottom). Exported CAZymes are those encoded by genes with predicted signal peptides. Black circles and vertical lines within violin plots represent the data mean and standard deviation, respectively. (**b**) Distribution of gene expression per CAZyme class overall and per capita.

Analyses of the estuarine metatranscriptome showed significantly greater CAZyme gene expression levels between sediment and water column communities across the estuary (Fig. 2a). This pattern was observed whether considering all CAZymes together (Wilcoxon test *P*-values < 0.05 at each site) or each CAZyme class individually (*P*-values < 0.05 except for PL at site 6 at 20–21 ppt salinity; Fig. S2 and Table S3). The difference was greatest at non-saline sites (sites 1 and 2 at 0.2–0.3 ppt salinity), where substantially higher expression was observed in sediment compared to water column samples for all CAZyme classes. Notably, higher expression in sediment versus water, regardless of salinity, was also observed for each CAZyme class after accounting for differences in genome abundance per site (genome coverage-normalized TPM; Fig. 2; Fig. S2). The same trend was observed for exported/secreted CAZymes (i.e., CAZyme genes with signal peptides) (Wilcoxon test *P*-values < 0.05 for each class except PL at site 6 and GT at sites 5 and 6, 21–24 ppt; Fig. 2a; Fig. S2). The exported fraction is more representative of the capacity to undertake the initial breakdown of large polysaccharides, given that most large polysaccharides require some extracellular initial processing to generate readily importable carbohydrate oligo- and monomers for intracellular utilization (26, 83, 84). These results support earlier experimental findings that demonstrated that the hydrolysis rates of several fluorescently labeled polysaccharides were much higher in marine sediment versus water (bottom water) (21).

Sediments can be highly heterogeneous habitats compared to the well-mixed or more structured water column (85, 86). Compositional heterogeneity observed in estuarine and marine sediments likely corresponds with “patchy” resource availability, in part influenced by sediment grain size, sediment permeability, and macrofaunal bioturbation (87–90). The limited connectivity of pore spaces in muddy sediments is also likely to impede the movement of resources and microbial cells, thus constraining resource accessibility. Further differences between water and sediment are likely due to substrate availability. Communities in the overlying water column are likely to actively consume labile carbohydrates, leaving behind microbial detritus and other recalcitrant substrates that fall toward the benthos or are exported to the ocean (43, 91). Therefore, we predict that sediment microbial communities need to have genetic mechanisms to utilize diverse forms of carbohydrates (represented by CAZyme gene richness), as results indicate here (Fig. 1b).

### CAZyme genes are more consistently transcribed in the water column

**Fig 3:**
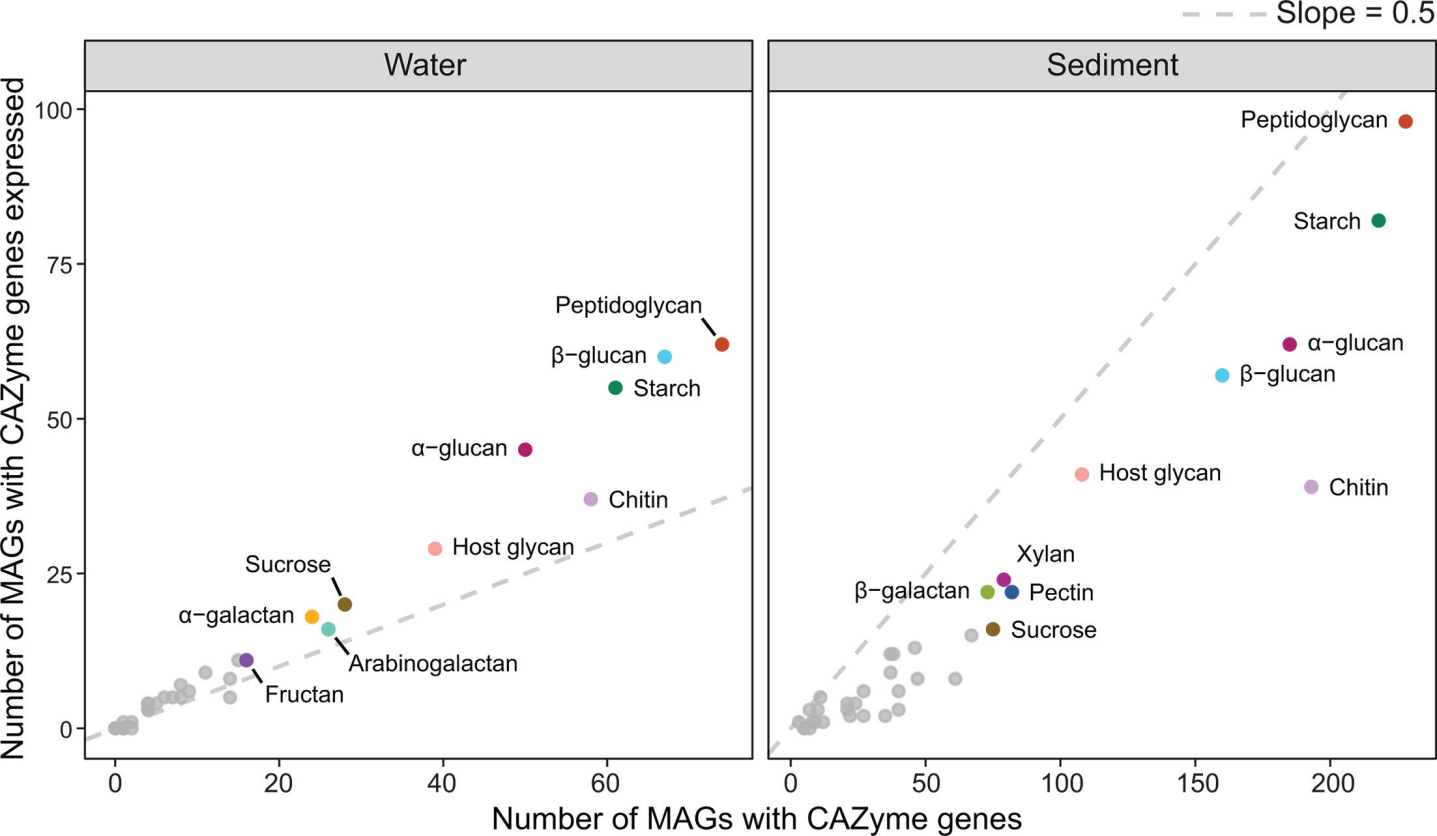
Substrate categories putatively targeted by CAZymes. The horizontal axis represents the number of populations (represented by MAGs) present per habitat that harbor CAZyme genes putatively targeting a given substrate (populations were deemed present if mapped reads covered ≥ 10% of the assembled genome length). The vertical axis represents the number of populations expressing those CAZyme genes in the habitat (i.e., ≥ 5 paired RNA reads mapped to the CAZyme gene in an MAG in a given sample). Colored points present the top 10 substrate categories based on MAG presence and expression. The dotted line has a slope of 0.5, where points above the line indicate that more than 50% of populations with CAZyme genes for a particular substrate are considered to have transcribed those genes and *vice versa*.

Despite higher overall CAZyme gene densities (Fig. 1b) and gene transcription in sediment communities (Fig. 2), planktonic populations were more likely to actively express their CAZyme gene repertoire. Collectively, MAGs recovered from the water column had CAZyme genes targeting 26 putative substrates (based on the dereplicated set of MAGs used for transcriptome read mapping). Planktonic taxa (95%) expressed at least one gene targeting carbohydrate substrates (versus 67% of sediment taxa). Furthermore, for 25 substrates, at least half the planktonic taxa with genes specific to each also transcribed those genes (Fig. 3, left panel). For example, 90% of taxa (i.e., MAGs) with genes for beta-glucan and 88% of taxa with genes for alginate metabolism exhibited associated transcriptional activity. Conversely, sediment communities had genes specific to 33 putative substrates, but less than half of taxa with genes targeting these substrates were found to express them (Fig. 3, right panel). This could indicate a greater capacity for opportunistic degradation of carbohydrates by sediment communities, consistent with the greater heterogeneity and resource patchiness of this habitat.

CAZyme gene family compositions largely overlapped across sediment and water column communities (Fig. 1a). Accordingly, we found a majority of the communities in both habitats actively expressed CAZyme genes associated with (i) recycling microbial biomass and cell growth (i.e., peptidoglycan) (92, 93); (ii) utilizing various structurally diverse N- and S-substituted host glycans and glycoconjugates (94); and (iii) degrading abundant structural polysaccharides (e.g., chitin and beta-glucan) (95) and storage polysaccharides (e.g., alpha-glucan, including starch) (96), as well highly labile sucrose (Fig. 3).

In addition to the substrate categories above, many members of the planktonic community were also found to express genes associated with the degradation of arabinogalactan, fructan, and ⍺-galactan (Fig. 3). Substrates such as arabinogalactan and fructan are commonly found in plants as constituents of structural and storage polysaccharides, respectively (97, 98). Meanwhile, the benthic community exhibited a preference for structural xylan, pectin, and β-galactan based on gene expression data (Fig. 3). Xylan and pectin are also structural components of plants, the former of which constitutes a major component of hemicellulose and is the second-most abundant polysaccharide after cellulose (80, 99). While we observed different putative preferences for ⍺-galactan degradation (planktonic preference) and β-galactan degradation (benthic preference), the specific CAZymes predicted were consistently exo-acting (e.g., β-galactofuranosidase EC 3.2.1.146, ⍺-galactosidase EC 3.2.1.22, and β-galactosidase EC 3.2.1.23). Galactose residues are common in marine substrates (both ⍺ and β forms) including carrageenan and agar (100) and terrestrial plant-based oligo- and polysaccharides such as rhamnogalaturonan, arabinogalactan, and raffinose (101).

Some of the differences in putative substrate preferences observed here likely reflect the relative roles of the substrate. For instance, arabinogalactan is a structural component of the plant cell wall and is involved in plant-microbe interactions (102, 103). Fructans can be synthesized by bacteria, where they constitute structural polysaccharides in biofilms, as well as by plants as storage polysaccharides and as a form of environmental stress response (104, 105). In an aquatic estuarine environment, the planktonic communities could be exposed to these substrates and capitalize on them as a nutrient source. In contrast, xylan and pectin are structural polysaccharides in plants that present incredibly complex structures consisting of diverse residues (28, 99). The complexity of these substrates likely requires time for the substrate to be broken down and utilized. Here, benthic communities may be better placed to compete for these resources.

### Genes of GH13 CAZymes that degrade alpha-glucans are ubiquitously transcribed

**Fig 4:**
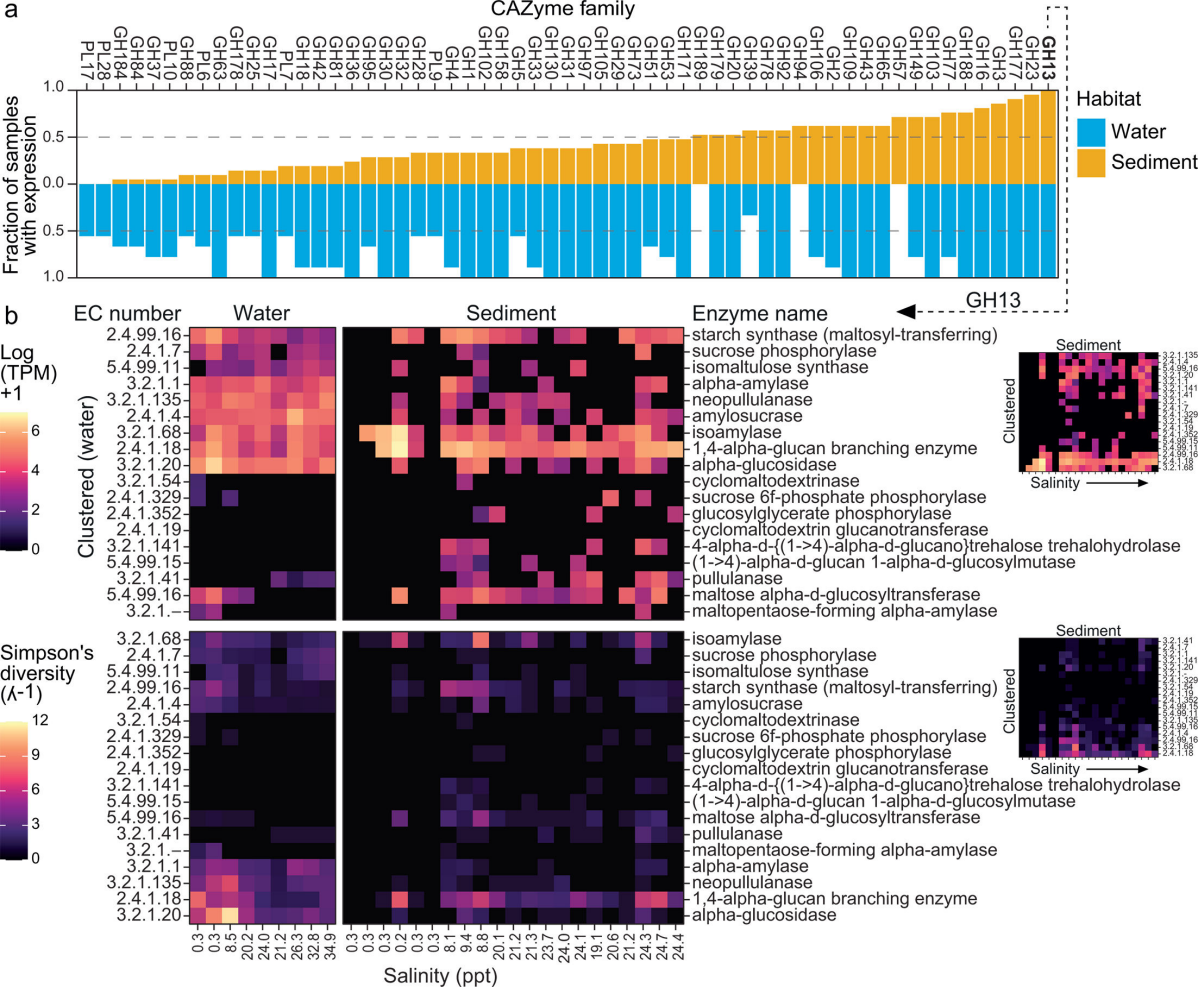
Ubiquitously expressed CAZyme gene families. (**a**) Fraction of samples in water and sediment across which each CAZyme gene family was expressed for those expressed in ≥ 50% of samples. (**b**) Upper plots: Summed TPM per site for GH13 CAZyme genes. Lower plots: Inverse Simpson’s diversity of genes expressed (TPM were summed per CAZyme gene per MAG). GH13 assignments were based on EC numbers. Vertical axes of the larger upper and lower plots (left side) show EC numbers ordered based on hierarchical clustering using Ward’s D2 method. Clustering was done based on only water samples with the vertical axes of sediment plots ordered to match (i.e., rows in water and sediment plots correspond to the same EC number and enzyme name, respectively). Smaller plots (right side) illustrate vertical axis clustering based on sediment samples. For both large and small heatmaps, samples along the horizontal axis are ordered from the first non-saline site 1 to the most saline site 9 from left to right. Sediment samples were collected in triplicate from the same site.

Out of all the CAZyme families, we observed that only genes associated with GH13 were expressed across the entire estuarine transect in both sediment and water column communities (Fig. 4a). The GH13 family consists of diverse CAZymes that target various ⍺-glucans including starch, glycogen, dextran (bacterial exopolysaccharide) (106), and pullulan (fungal exopolysaccharide) (107). While classified as GH, CAZymes in GH13 have been shown to also have other activities related to alpha-glucan degradation and synthesis such as hexosyltransferases (EC 2.4.1.-) and intramolecular transferases (isomerases; EC 5.4.-.-). Throughout the salinity gradient, we observed that communities in the water column transcribed more GH13 CAZyme genes associated with alpha-glucan degradation, such as those encoding alpha-amylase, alpha-glucosidase, isoamylase, and neopullulanase, whereas sediment prokaryotic communities were more likely to transcribe genes encoding biosynthetic GH13 CAZymes such as starch synthase and 1,4-alpha-glucan branching (glycogen) enzyme (Fig. 4b), suggesting that sediment bacteria stockpile carbohydrates as a survival strategy in patchy habitats. Transcription of hydrolase genes was spatially heterogeneous in sediment.

Additionally, we estimated the level of active competition for substrate degradation (or functional redundancy for substrate biosynthesis) via alpha diversity of transcribed CAZyme genes. Using this approach, competition in both habitats was highest for starch (inferred based on Simpson’s diversity of isoamylase genes expressed in water and sediment and alpha-amylase genes in water) and was additionally high in water for pullulan (neopullulanase genes) and oligosaccharides (alpha-glucosidase genes) (Fig. 4b). However, the degree of putative competition appeared to be dependent on location in the estuary, whereby competition for these alpha-glucans was highest at low salinities in water (non-saline and brackish sites at 0.3–8.5 ppt salinity). In sediment, functional redundancy for alpha-glucan biosynthesis was similarly highest at the transitional low-salinity brackish site (site 3, 8.1–9.4 ppt salinity). Higher taxa richness generally equates to higher functional diversity and redundancy (81, 108). This potentially explains the higher estimated redundancy for alpha-glucan degradation and biosynthesis in sediment at the transitional brackish sediment site, where a substantially greater number of MAGs were recovered compared to any other sediment site (at least twice as many, Fig. 1a). The number of MAGs recovered from water was comparable across sites, suggesting other factors, such as substrate influx from riverine sources, were responsible for the difference in alpha-glucan competition between non-saline and saline parts of the water column.

### Planktonic prokaryotic communities compete for beta-1,3-glucans

**Fig 5:**
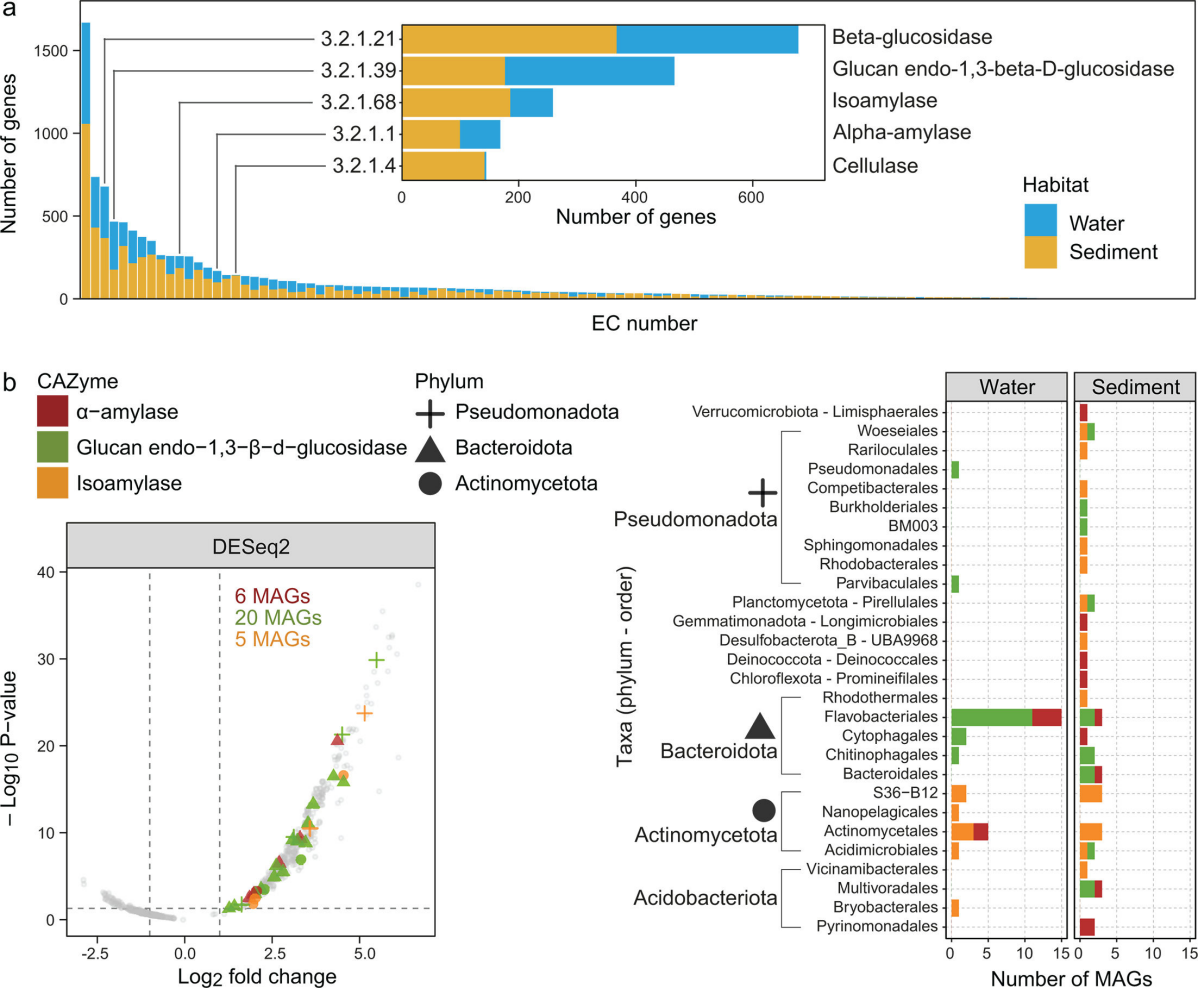
Gene expression associated with beta-glucan degradation. (**a**) CAZymes organized by EC number. EC numbers are ranked (x-axis) based on the number of genes assigned to them. Colors represent the habitat where MAGs were recovered from. The inset shows the top four glucan-targeting endo-acting CAZymes (glucan endo-1,3-beta-D-glucosidase, isoamylase, alpha-amylase, and cellulase) with beta-glucosidase for reference. (**b**) Left: Volcano plot of differential expression analyses of all CAZymes with EC assigned. Log fold change indicates greater expression in water (positive values) versus greater expression in sediment (negative values). Input data were summed TPM per CAZyme EC per MAG. *P*-values on the vertical axis were adjusted using the false discovery rate method. Right: Number of MAGs grouped by phyla and order that expressed endo-1,3-beta-D-glucosidase, isoamylase, and alpha-amylase genes in any sample.

Among the top substrates putatively degraded in the sediment and water column were beta-glucans. In the water column, a majority of taxa expressed CAZyme genes that are predicted to catalyze the degradation of beta-glucans, second only to peptidoglycans (Fig. 3). While cellulose is the most abundant and recognizable form of beta-glucan polysaccharide (109), its crystalline form is not easily degradable without a suite of enzymes (110). A ranking of EC numbers based on the number of genes recovered showed cellulase genes were common in sediment, but largely lacking from taxa in the water column (Fig. 5a), reflecting the greater capacity of benthic communities to degrade settled recalcitrant organic matter potentially derived from mangroves in the intertidal zone or terrestrial plants adjacent to the estuary.

Glucan endo-1,3-beta-D-glucosidase (EC 3.2.1.39) genes were especially prevalent in the water column (Fig. 5a). Results from differential expression analyses using DESeq2, edgeR, and limma all agree that the expression of genes encoding for this CAZyme was significantly higher in the water column and that greater numbers of MAGs expressed these genes compared to those for other endoglucanases such as alpha-amylases and isoamylases (Fig. 5b, and method comparison in Fig. S3). Glucan endo-1,3-beta-D-glucosidase catalyzes the endo-hydrolysis of beta-1,3-glucans such as bacterial curdlan (an exopolysaccharide), paramylon from euglenozoans, and algal laminarin (111). These substrates are naturally abundant in aquatic habitats. In the marine environment, laminarin (β−1,3-linked with β−1,6 branches) is the predominant form of β-glucan (11). Previous work by our group detected an abundance of active photosynthetic cryptophytes in the water column and diatoms in the benthos in the non-saline regions of the estuary (10). The main storage polysaccharide in cryptophytes is starch (α−1,4-linked with α−1,6 branches), whereas diatoms typically store chrysolaminarin (a laminarin-like polysaccharide) in vacuoles (112). While both types of storage polysaccharides are considered to be easily accessible and degradable substrates, they vary in their solubility in water. For example, starch is often found as granules, with a higher ratio of branching increasing their solubility (by reducing granule packing density) and degradability (113). As for starch, chrysolaminarin and laminarin (or laminarins) can be insoluble or soluble (113–115), with the solubility increasing with the degree of branching (114), although laminarins tend to be regarded as highly soluble (11, 116). Here, fluvial and tidal forces mixing and distributing chrysolaminarin from the benthos into the overlying water column could, in part, explain the wide transcriptional distribution of genes encoding glucan endo-beta-1,3-glucosidase in the water column.

**Fig 6:**
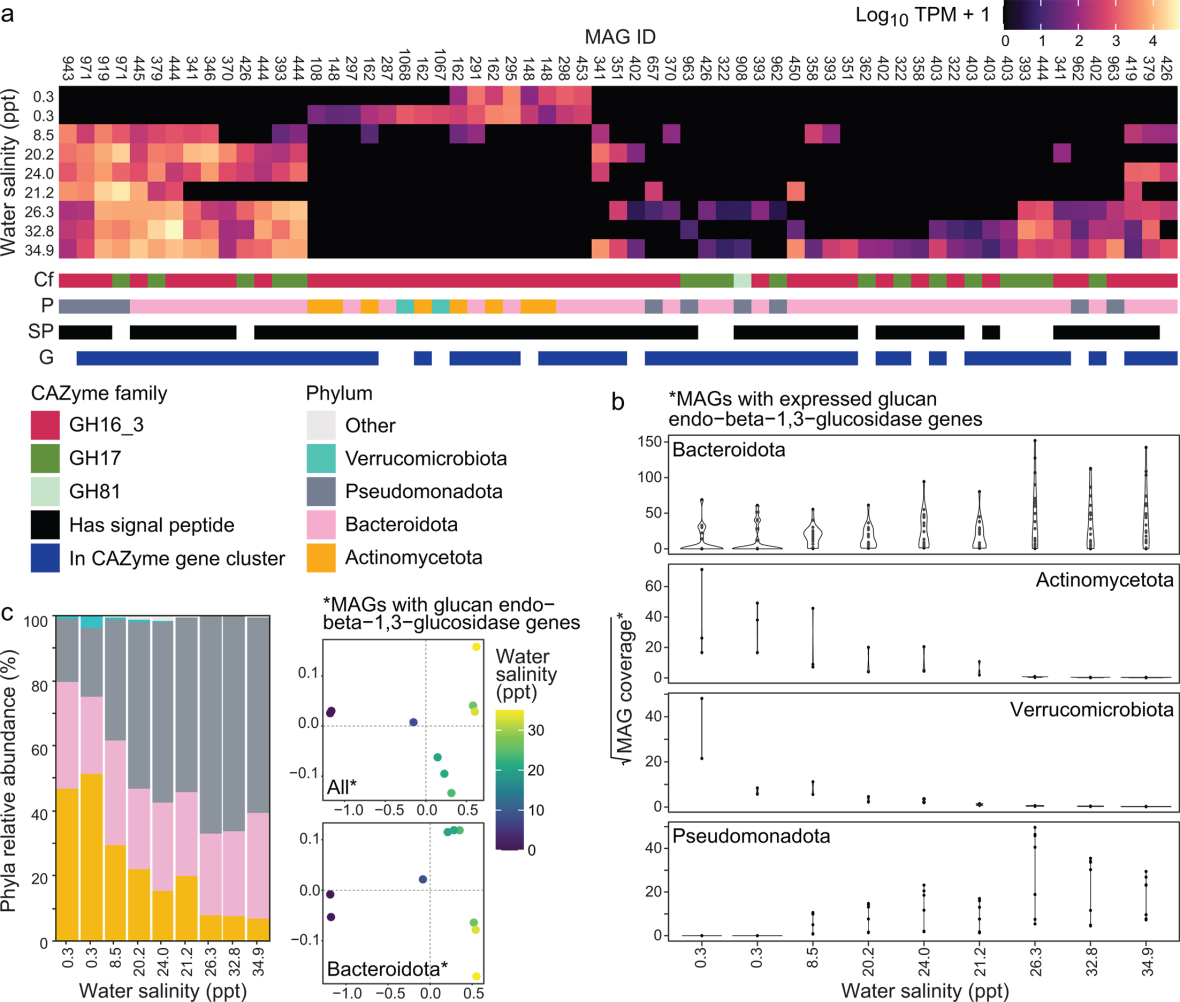
Expression of glucan endo-1,3-beta-D-glucosidase genes and abundance of associated phyla. (**a**) Heatmap at top shows the expression of glucan endo-1,3-beta-D-glucosidase (EC 3.2.1.39) genes across all water column samples (TPM). Columns with the same MAG IDs represent genes from the same MAG. Heatmap rows represent sampling sites 1 to 9 with increasing salinity (downward). Additional tracks (rows) beneath the main heatmap represent CAZyme families (Cf), phyla (P), the presence of signal peptides (S, black = present), and the inclusion of the gene in a CAZyme gene cluster (G, blue = included in CGC). (**b**) Distribution of MAGs by phyla that putatively expressed glucan endo−beta−1,3−glucosidase across the salinity gradient in water. (**c**) Left: Relative abundance of phyla associated with endo-1,3-beta-D-glucosidase gene expression in water. Right: Beta diversity of taxa in water with glucan endo-1,3-beta-D-glucosidase genes (top: taxa from all four phyla; bottom: Bacteroidota only). Ordinations are based on Bray-Curtis dissimilarities of MAG relative abundance.

Further analysis of the expression pattern of glucan endo-1,3-beta-D-glucosidase-encoding genes showed partitioning according to the salinity based on the phylum and CAZyme class. Bacteroidota expressed these genes regardless of the salinity, while the expression by other phyla was associated with distinct habitat salinities: Actinomycetota and Verrucomicrobiota in non-saline water predominantly and Pseudomonadota exclusively in saline water (Fig. 6a, track P, and Fig. 6b). These patterns in expression reflect the distributions of Bacteroidota and Verrucomicrobiota in the water column (Fig. 6c). Bacteroidota was one of the most abundant and prevalent phyla, and Verrucomicrobiota, while comparatively rare, was most abundant in non-saline water. They are also consistent with the well-known capacity of Bacteroidota to degrade complex organic carbon (117). Alongside Bacteroidota, Actinomycetota and Pseudomonadota were the other two most abundant and prevalent phyla. Despite their prevalence, all three phyla exhibited differences in taxonomic composition between non-saline and saline parts of the estuary (Fig. 6c; Table S1). These compositional differences likely explain the niche differentiation in glucan endo-1,3-beta-D-glucosidase gene expression exhibited by Actinomycetota and Pseudomonadota.

The majority of Bacteroidota expressing glucan endo-1,3-beta-D-glucosidase belonged to the order Flavobacteriales (Fig. 5b), a lineage known for its extensive capabilities for carbohydrate degradation (118) and wide environmental distribution (119). Endo-1,3-beta-D-glucosidase gene expression in non-saline water was undertaken by members of the family Microbacteriaceae (Actinomycetota) and order Opitutales (Verrucomicrobiota). Members of the former are primarily found in freshwater (120–122), although different members of this family were present across the entire estuarine salinity gradient in the sampled estuary (53); the latter (Opitutales) have been detected in diverse aquatic (123), terrestrial (124, 125), and host-associated habitats (126). Finally, members of the Pseudomonadota that expressed glucan endo-beta-1,3-glucosidase genes in saline water were from the orders Pseudomonadales and Parvibaculales, both of which are abundant in other coastal environments (127–129).

The expression of glucan endo-beta-1,3-glucosidase genes was also partitioned according to CAZyme families, where the gene expression of GH17 was found only in saline water, and GH16_3 was found across the salinity regime (Fig. 6a). Taxa in saline water were often found to encode for and express genes associated with both families. These findings are congruent with current knowledge about the ecological distribution of these CAZyme families. To date, all characterized bacterial GH17 glucan endo-beta-1,3-glucosidase listed in the CAZy database have been derived from marine genera such as *Muricauda* (130), *Formosa* (41, 131), and *Vibrio* (132). Meanwhile, GH16 was characterized in bacteria isolated from diverse environments, including soil (133–136), hot springs (137, 138), arctic sediment (139), human gut (140), and the ocean (141–145).

**Fig 7:**
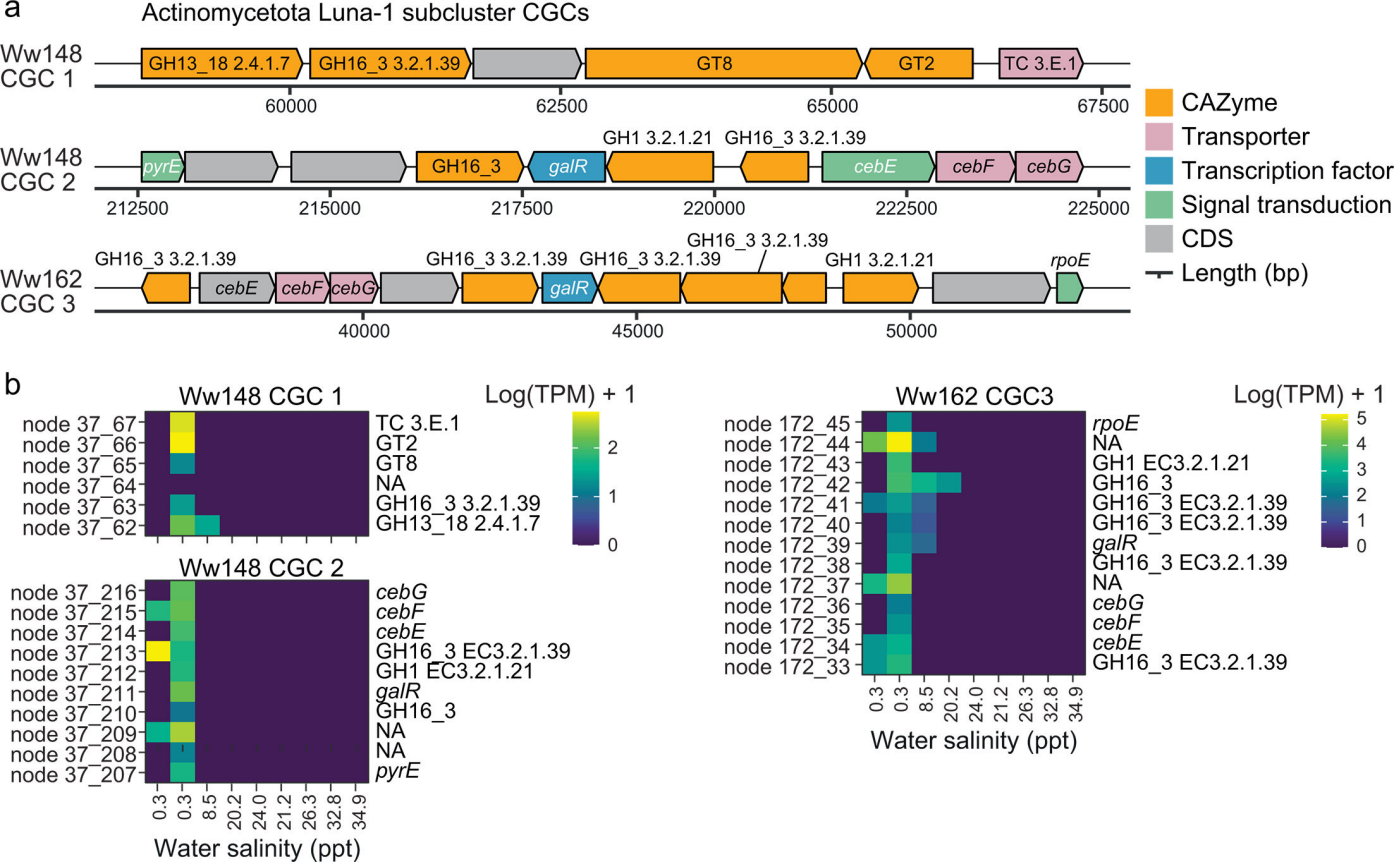
CGCs associated with active expression of glucan endo-1,3-beta-D-glucosidase genes in Luna-1 subcluster populations. (**a**) Gene maps showing organization of genes in the CGCs. The CGCs are from MAGs Ww148 and Ww162. (**b**) Expression of genes in each CGC across the salinity gradient in water is given as TPM. Contig/node and gene numbers (format node_gene number) are shown on the left vertical axis of each heatmap, and gene annotations are shown on the right.

CAZymes often work together to degrade substrates, and genes encoding for CAZymes are often colocated with other CAZyme genes or genes that encode for transcription factors, signal transduction proteins, and transporters (62). These genetic structures can be predicted from genome assemblies and are known as CGC. We found that most of the glucan endo-beta-1,3-glucosidase genes were part of CGCs and that most CGCs belonged to MAGs from the Bacteroidota lineage (Fig. 6a, track G). This finding was expected as many members of the Bacteroidota contain CGCs known as PULs (146, 147). These PULs are characterized by the presence of *susCD* gene pairs (146, 147), which encode starch-binding proteins (148). Of these, SusC is also thought to be important for the uptake of glycan degradation products (149, 150). Accordingly, 203 *susCD* gene pairs were identified in Bacteroidota PULs in this study (Table S4). CGCs with glucan endo-beta-1,3-glucosidase genes were also found in Pseudomonadota and Actinomycetota MAGs. An unexpected finding was the presence and expression of three beta-1,3-glucan targeting CGCs in the Microbacteriaceae Luna-1 subcluster of Actinomycetota (*Rhodoluna* populations MAGs Ww148 and Ww162) (Fig. 7), along with another 10 CGCs in these two populations targeting substrates such as alpha-galactan, starch, and host glycans (where at least one GH or PL gene was expressed (Table S5). CGC presence in Actinomycetota is, in general, poorly explored, although analysis of the dbCAN database showed Actinomycetota contain 9% of all known bacterial CGCs (62).

Of the three CGCs associated with beta-1,3-glucan gene expression in the Luna-1 subcluster, the beta-1,3-glucan targeting CGC (CGC3) in MAG Ww162 was predicted to contain four GH16 glucan endo-beta-1,3-glucosidases genes (annotated as EC 3.2.1.39) along with genes encoding for ATP-binding cassette transporters specific to cellobiose (products of beta-1,3-glucan depolymerization), a LacI family transcriptional regulator, an RNA polymerase sigma-70 factor (signal transduction), and a GH1 beta-glucoside (Fig. 7; Table S5). We also detected two CGCs that putatively target beta-1,3-glucans in MAG Ww148 (CGC1 and CGC2), one of which CGC2 had a similar composition to that of Ww162, albeit with fewer copies of GH16 endo beta-1,3-glucosidase gene. Genes within all three CAZyme gene clusters were actively transcribed in non-saline water (Fig. 7; Table S5), indicating the capacity for coordinated gene expression of carbohydrate degradation in the actinomycetota Luna-1 subcluster. Members of the Luna-1 subcluster are photoheterotrophs (53, 151) with small, streamlined genomes (53). Previous analysis of the Luna-1 subcluster by our group showed both *Rhodoluna* taxa (represented by MAGs Ww148 and Ww162) were relatively most transcriptionally active and abundant in non-saline water and indicated that some members of the subcluster, particularly those from non-saline water, could degrade various substrates from hemicellulose and algal sources, including via beta-1,3-glucans (53). Results here further suggest that they can utilize coordinated gene expression to efficiently deploy degradative machinery in the same way as Bacteroidota with vastly larger genome sizes and different lifestyles and metabolisms (117, 152).

### Conclusions

Most studies to date evaluate carbohydrates in the environment by their size and general properties (e.g., solubility, bioavailability, and degree of microbial assimilation). Here, we present finer-grained genome-based analysis of putative polysaccharides degraded by the prokaryotic community differentiated by salinity and habitat (i.e., water column versus sediment). Our findings reveal a richer CAZyme-encoding gene repertoire in estuarine sediments than water, suggesting broader metabolic “preparedness” for diverse substrates. Findings further suggest a higher fraction of transcription in sediment than water is associated with carbohydrate degradation, consistent with the findings of prior experimental work (21). Comparatively, carbohydrate-degrading prokaryotes in the water column, while less diverse in the CAZyme-encoding gene repertoire, were more likely to express their degradative genetic machinery. While the taxonomic composition of organisms was different between salinity zones, we found that genes involved in degrading substrates such as alpha-glucans (e.g., starch and glycogen) and beta-1,3-glucans (e.g., laminarin) were ubiquitous. Our results suggest that the estuarine prokaryotic community actively degraded autochthonous organic carbon (e.g., microbial biomass such as chrysolaminarin, starch granules, and peptidoglycans) and more complex autochthonous and allochthonous organic carbon (e.g., mangrove and terrestrial plant biomass such as cellulose and hemicellulose, particularly in the non-saline and brackish zones). Our findings suggest that water column communities are likely to assimilate much of the easily degradable substrates such as starch and laminarins, leaving behind larger and more complex substrates such as xylans and arabinans (both part of hemicellulose) to be degraded by prokaryotic communities in sediments. Finally, we report the presence and expression of CGCs putatively targeting beta-1,3-glucans in all three dominant and prevalent estuarine phyla (Bacteroidota, Pseudomonadota, and Actinomycetota), including the Actinomycetota Luna-1 subcluster. Future work will need to assess the substrate specificity of the predicted enzyme functions found in the community. Furthermore, carbohydrate degradation is known to involve collaboration by multiple taxa. Another avenue for exploration is the degree of division of labor between different taxa in their utilization of complex substrates.

## Data Availability

Sequence data were deposited under NCBI BioProject PRJNA668816. Data tables used for analyses are available here: https://doi.org/10.5281/zenodo.17709343.

## SUPPLEMENTAL MATERIAL

Supplemental figures aem.01852-25-s0001.pdf Figures S1 to S3.

Supplemental tables aem.01852-25-s0002.xlsx Tables S1 to S5.

ASM does not own the copyrights to Supplemental Material that may be linked to, or accessed through, an article. The authors have granted ASM a non-exclusive, world-wide license to publish the Supplemental Material files. Please contact the corresponding author directly for reuse.
