# Supplementary material for: Genetic mechanisms for estuarine carbohydrate degradation and linked transcriptional activity
Source: Appl Environ Microbiol. 2026 Jan 13;92(2):e01852-25. doi: 10.1128/aem.01852-25 (PMC12915301; doi:10.1128/aem.01852-25)
Supplement: Supplemental figures — Figures S1 to S3. [file aem.01852-25-s0001.pdf]

Supplementary Figures

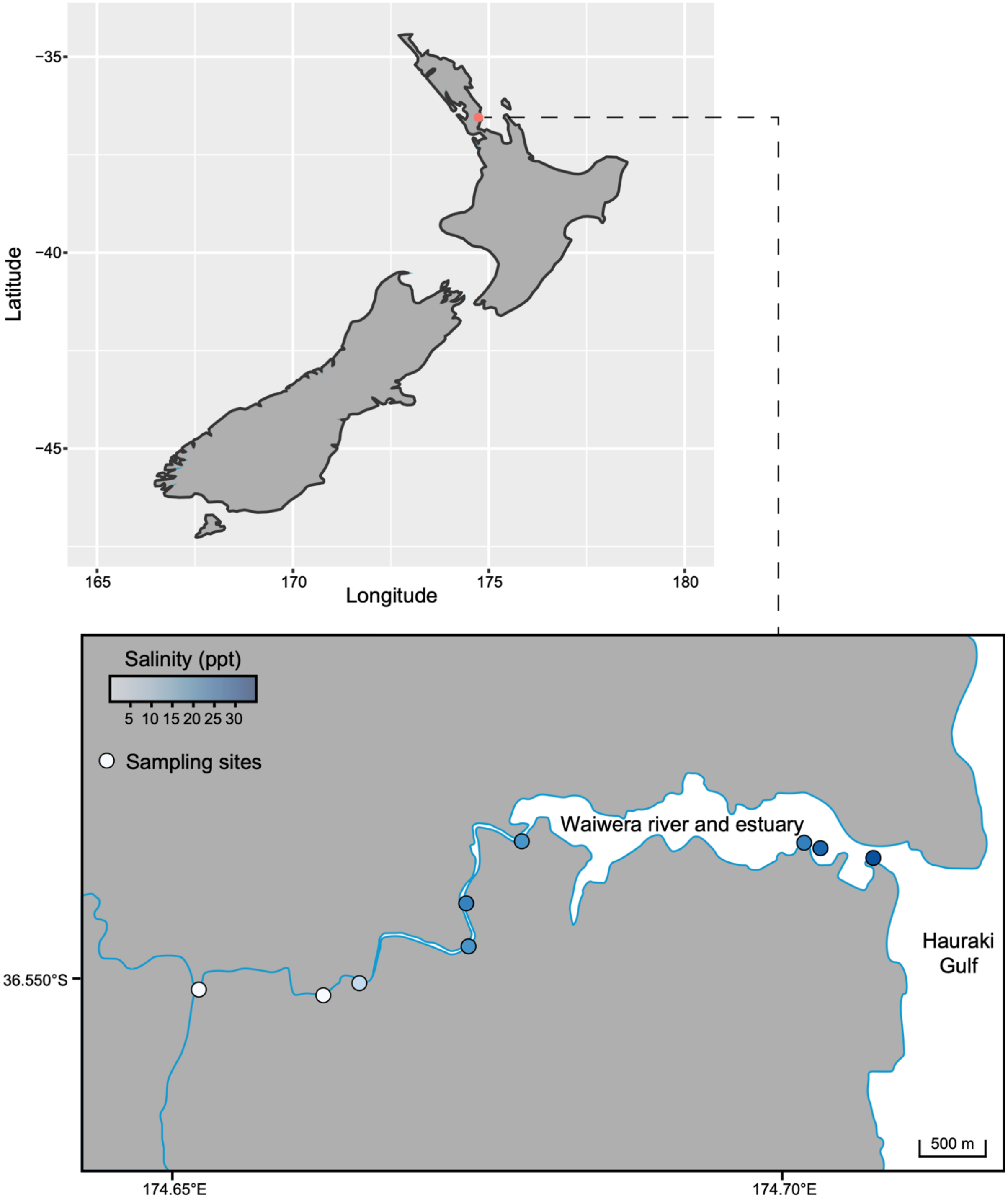

Supplementary Figure S1 Maps showing sampling locations. Upper map: location of the Waiwera river and estuary in New Zealand. Lower map: sampling sites along the river and estuary system across a freshwater to marine. Sampling sites were designated 1 to 9 from left to right with site 9 at the estuary mouth where water flows into the Hauraki Gulf.

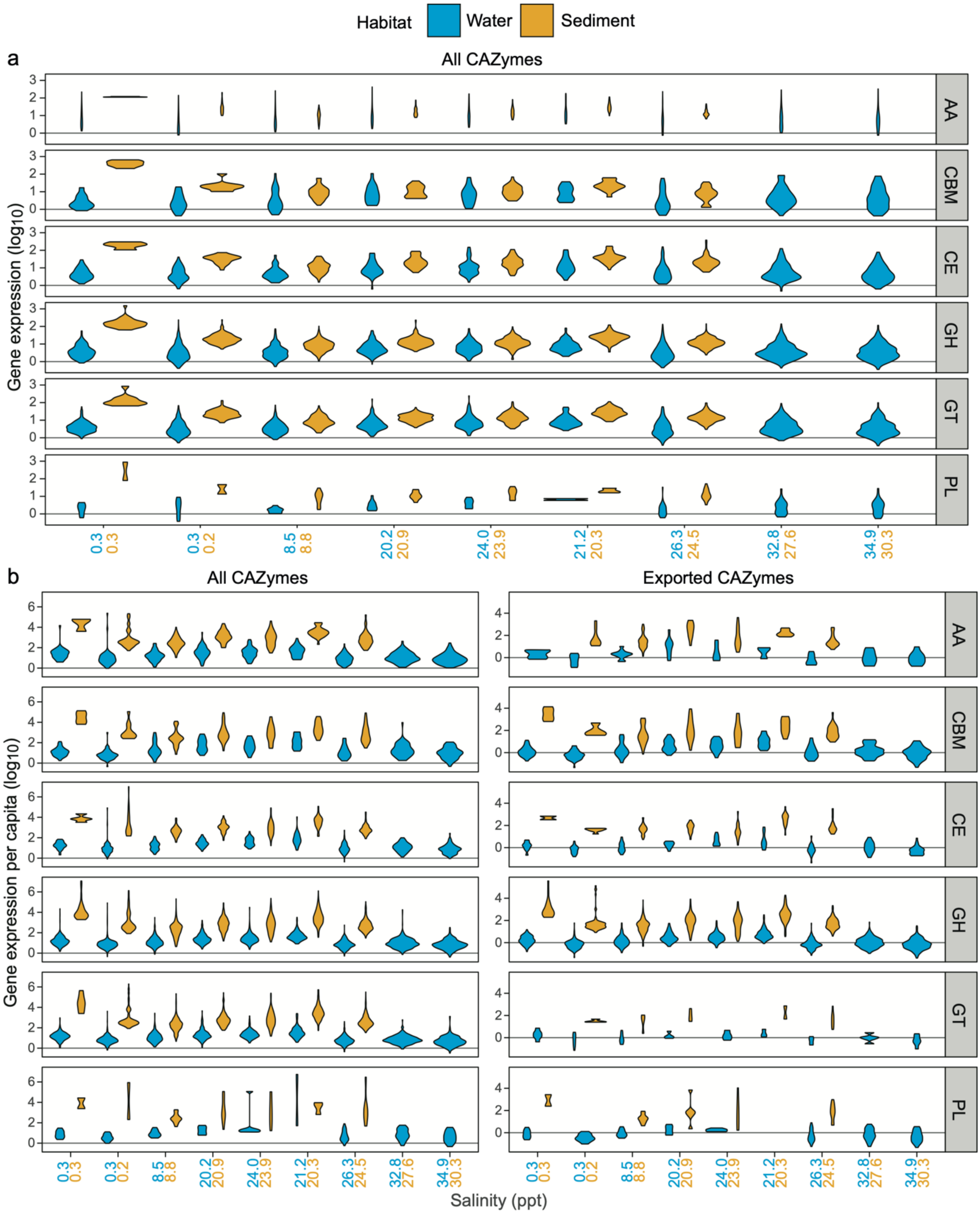

Supplementary Figure S2 Gene expression across all samples per CAZyme class per MAG. Distribution of gene expression (TPM) in the estuary overall (top) and after normalising to genome coverage (expression per capita) for all genes (bottom left) and the genes of exported CAZymes (bottom right). Exported CAZymes are those encoded by genes with predicted signal peptides.

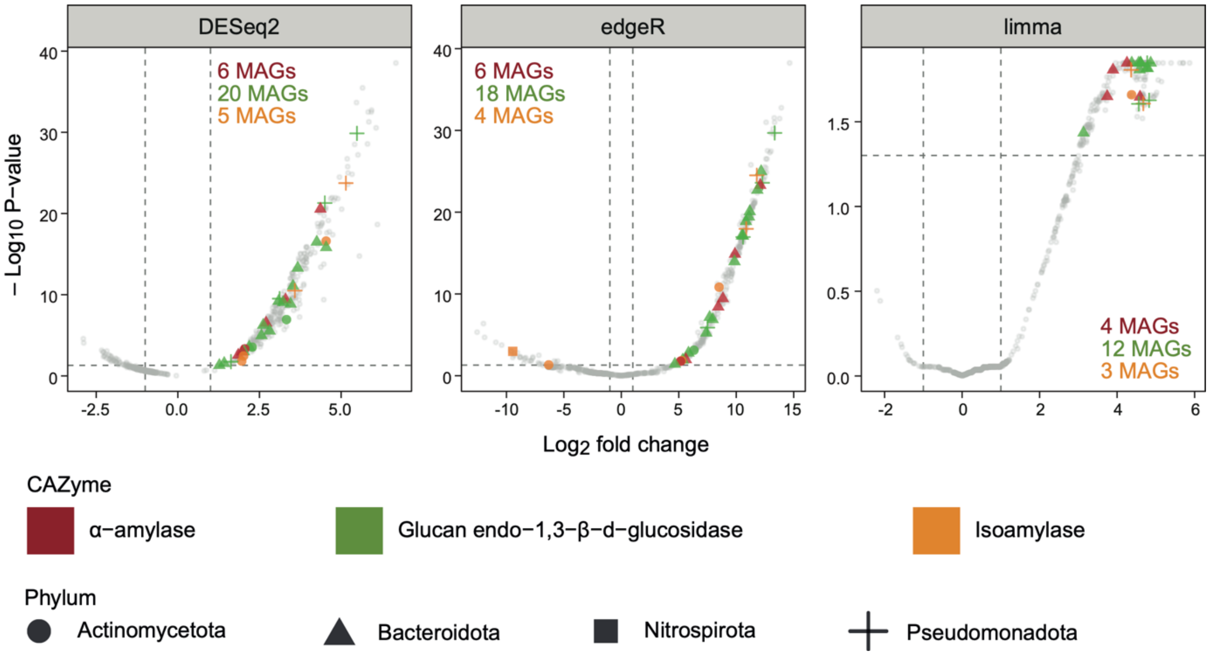

Supplementary Figure S3 Comparison of differential gene expression associated with beta-glucan degradation based on DESeq2, edgeR and limma. Volcano plots of differential expression analyses of all CAZymes with EC assigned. Log fold change indicates greater expression in water (positive values) versus greater expression in sediment (negative values). Input data were summed TPM per CAZyme EC per MAG. P-values on the vertical axis were adjusted using the false discovery rate method.
